# Antibody immunoglobulin G1 and immunoglobulin G2a responses against some cystic fluid proteins of *Cysticercus bovis* in Balb/c mice

**DOI:** 10.14202/vetworld.2018.1641-1647

**Published:** 2018-11-30

**Authors:** I Nyoman Mantik Astawa, Ida Bagus Made Oka, I Made Dwinata

**Affiliations:** 1 Laboratory of Immunology, Faculty of Veterinary Medicine, Udayana University, Denpasar Bali 80232, Indonesia; 2 Laboratory of Parasitology, Faculty of Veterinary Medicine, Udayana University, Bali 80232, Indonesia

**Keywords:** Cystic fluid, *Cysticercus bovis*, immunoglobulin G1, immunoglobulin G2a, proteins

## Abstract

**Background and Aim:**

Immunoglobulin (Ig) G1 and IgG2a are the surrogate markers respectively for humoral and cellular immune responses of hosts against antigens including cystic fluid proteins of *Cysticercus bovis*. A study was conducted to investigate the IgG1 and IgG2a responses of Balb/c mice against some individual cystic fluid proteins of *C. bovis* in an effort to determine the roles of each protein in inducing the humoral and cellular immune responses in host.

**Materials and Methods:**

Individual p71, p31, and p14 proteins of *C. bovis* were purified by separation of the proteins using sodium dodecyl sulfate-polyacrylamide gel electrophoresis and elution of individual proteins from the gel. Six female Balb/c mice were immunized 4 times at 10-day intervals with the crude cystic fluid proteins, and sera were collected for the measurement of IgG1 and IgG2a levels against the individual proteins. Sera samples collected before the first immunization were used as negative antibody control, sera samples collected after the fourth immunization were used as positive antibody control, and crude cystic fluid protein was used as positive antigen control.

**Results:**

All immunized mice were immune to p71, p31, p14, and crude cystic fluid proteins of *C. bovis*. The crude cystic fluid proteins of *C. bovis* induced a higher IgG2a than IgG1 level following the first and the second immunizations but switched into a higher IgG1 than IgG2a level following the fourth immunization. Protein 71 kDa (p71) induced a higher IgG2a than IgG1 level following the fourth immunization. In contrast, p14 induced a higher IgG1 than IgG2a level following the fourth immunization. Low and balance IgG1 and IgG2a levels against p31 were observed following the first to the fourth immunizations.

**Conclusion:**

Using IgG1 and IgG2a levels as the surrogate markers, it appears that cystic fluid antigens of *C. bovis* induce both humoral and cellular immune responses in Balb/c mice. The p71 appears to be a better inducer of cellular immune response, whereas p14 is a better inducer of humoral immune response of mice.

## Introduction

Cysticercosis caused by *Cysticercus bovis*, the larval stage of *Taenia saginata*, is still very common parasitic infection among cattle population worldwide. The parasites can persist for months to years in tissues such as cardiac and skeletal muscles, liver, lungs, kidneys, and lymph nodes [1]. In general, the presence of the parasites such as *Cysticercus* in a host for prolonged periods induces both cellular and humoral immune responses. Life parasites are generally weak inducers of immune responses as they can evade the host immune system [2]. The dead or dying parasites can, however, induce both cellular and humoral immune responses in the infected hosts [3]. In human, granulomatous inflammatory immune response induced by *Cysticercus cellulosae* causes tissue injury and contributes to the clinical signs of the disease [4]. In a murine animal model with *Taenia crassiceps cysticercus*, cellular immune response occurs in the early stage of infection characterized by the increase of interferon gamma (IFN-γ) and interleukin (IL)-2 levels. In the later stage, however, it switches to humoral immune response indicated by the progressive increase of IL-4 production [5].

Host cellular and humoral immune responses play important roles in controlling parasitic infection. Parasitic antigen-antibody complex bound to the Fc receptors (FcRs) on the surface of innate immune cells such as basophils, eosinophils, mastocytes, monocytes, and macrophages can trigger antibody-dependent cells cytotoxicity response [6]. In addition, IgE-antigen complex bound to FcRs on the surface of mastocytes can induce degranulation and the release of mediators such as histamine which is capable of inhibiting parasites activities in tissues [7]. Studies on immune response against the antigens of *C. bovis* in cattle and the roles of cysticercus proteins in host immunity are still very limited. In *C. cellulosae*, the larval stage of *Taenia solium* in human, antibodies against somatic, cyst wall, and cyst fluid antigens have been detected in the infected human [8,9]. In the infected cattle, antibody against 260 kDa, 150 kDa, 130 kDa, 67 kDa, 60 kDa, 55 kDa, 50 kDa, 23 kDa, 18 kDa, and 14 kDa proteins of *C. bovis* has also been detected [10]. Cystic fluid proteins of *C. bovis* were also able to induce an antibody response in mice [11]. At present, three proteins (14 kDa, 31 kDa, and 71 kDa) of *C. bovis* have been purified, and their roles in host immune responses were investigated using Balb/c mice as an animal model.

The types of host immune responses against pathogens can be identified using immunoglobulin (Ig) isotypes (IgM, IgG1, IgG2a, IgG2b, IgG3, and IgE) as surrogate markers. In mice, IgG1 and IgE have been widely used as the surrogate markers of humoral antibody (T-helper [Th2] activation) responses as IL-4 secreted by Th2 cells induces Ig class switching into IgG1 and IgE subclasses [12]. IL-4 plays an important role in antibody production by inducing the proliferation and differentiation of B cells into plasma cells. On the other hand, IgG2a and IgG3 are the surrogate markers of cellular immune response (Th1 activation) as IFN-γ produced by Th1 cells induces Ig class switching into IgG2a or IgG3 subclasses [13]. Th1 cells produce IFN-γ and IL-2 which induce activation of both innate and adaptive cellular immune responses [14] such as activation of natural killer (NK) cells [15], CD8^+^ T cells [16], and macrophages [14].

In this study, therefore, the roles of *C. bovis* individual proteins (p71, p31, and p14) in host immune responses were investigated by determining IgG1 and IgG2a responses of against those three proteins using Balb/c mice as experimental animals.

## Materials and Methods

### Ethical approval

This study has been approved by the Ethical Commission for the Use of Animals in Research and Education of the Faculty of Veterinary Medicine, Udayana University, Bali, Indonesia, with Ethical Clearance No. 350/KE-PH/H/2018.

### Preparation of C. bovis crude proteins

Crude proteins of *C. bovis* were obtained from two Bali Cattle experimentally infected with gravid proglottids of adult *T. saginata* containing approximately 500,000 oncospheres per cattle [11]. The cysticerci developed in the infected cattle were collected from skeletal and visceral organs at day 103 (one cow) and 131 (one cow). The cysts were then cut into pieces and suspended in phosphate-buffered saline (PBS). Following centrifugation at 1000 × g for 10 min, the supernatant fluid was collected and stored at −70°C until used.

### Immunization of Balb/c mice

Six of 7-week-old female mice were immunized intraperitoneally 4 times at 10-day intervals with 0.2 ml crude cystic fluid antigen (containing of approximately 10 μg protein) emulsified in complete Freund’s adjuvant (the first immunization), in incomplete Freund’s adjuvant (the second and the third immunizations) and without adjuvant (the fourth immunization). Blood samples were collected from orbital sinus 7 days before the first immunization and 7 days following each immunization. The collected blood samples were stored at 4°C for 18 h, and sera were then collected by centrifugation at 1000 × g for 5 min. Sera samples were then transferred into Eppendorf tubes and stored at −20°C until used. Pooled sera collected before immunization were used as negative antibody control and those collected 7 days following the fourth immunization were used as positive antibody control. The immune response of each mouse was then tested by enzyme-linked immunosorbent assay (ELISA) as described below.

### Purification of cystic fluid individual proteins

Individual cystic fluid proteins of *C. bovis* were purified by elution of the proteins from sodium dodecyl sulfate-polyacrylamide gel electrophoresis (SDS-PAGE) gel blindly cut into 15 pieces. The cystic fluid was first clarified by centrifugation at 7000 × g for 10 min and was then subjected to SDS-PAGE [17] using large gel electrophoresis system (TV400Y Standard Twin-Plate Maxi-Gel Electrophoresis Units Sci-Plus, UK). The gel used was at the concentration of 10% and was then cut horizontally into 15 pieces. Each gel cut was homogenized in a mortar and suspended in 3 ml elution buffer (5 mM dithiothreitol, 50 mM Tris-HCl pH 7.9, 0.1% SDS, 0.15 M NaCl, 0.1 mM ethylenediaminetetraacetic acid, and 1 mM phenylmethylsulfonyl fluoride). After incubation with shaking for overnight at 4°C, the supernatant was collected by centrifugation at 1000 × g for 5 min and precipitated with acetone (1 elute:4 acetone) [18]. The precipitate was collected by centrifugation at 1000 × g for 5 min, air-dried, and diluted in PBS pH 7.4.

The eluted individual proteins were tested by ELISA using polyclonal antibody (pAb). ELISA microtiter plate was first coated for overnight with crude cystic fluid proteins (5 μg/ml) or with purified individual proteins (1 μg/ml) diluted in coating buffer (50 mM Na_2_CO_3_, 50 mM NaHCO_3_, and pH 9.6) for overnight at 4°C and washed twice with PBS-T (PBS containing 0.1% Tween 20). The microplate was then blocked for 1 h at 37°C using 5% skim milk in PBS. After 3 times washes with PBS-T, a volume of 100 µl of pooled pAb diluted 1/500 with 3% skim milk in PBS-T was added into each well and incubated at 37° for 1 h. 100 µl anti-mouse IgG-horseradish peroxidase (HRP) (KPL, USA) diluted 1:1000 in PBS-T containing 1% skim milk was added into each well and incubated for 1 h at 37°C. Wells of the ELISA plate were washed 4 times with PBS-T and 100 µl 3,3′,5,5′-tetramethylbenzidine (TMB) substrate (KPL, USA) was added into each well. Following incubation at room temperature for 15 min, 50 µl stop solution (1 N H_2_SO_4_) was added into each well, and the optical density (OD) of the substrate in each well was read using ELISA microplate reader using 450 nm filter.

The ELISA positive elutes were then subjected to Western blotting assay as described by Dunn [19]. Samples of ELISA positive elutes and crude cystic fluid proteins were first subjected to SDS-PAGE analysis using Mini Protean tetra cell (Bio-Rad, USA) according to the procedure as described by Laemli [14]. The proteins in the gel were then transferred onto nitrocellulose membrane by Mini Trans-Blot cell using carbonate-bicarbonate transfer buffer (10 mM NaHCO3, 3 mM Na_2_CO_3_, pH 9.9, and 20% methanol). Following 1 h blocking at room temperature with 5% skim milk in Tris-buffered saline (TBS/100 mM Tris-HCl pH 7.4), the nitrocellulose membrane was then soaked in anti-*C. bovis* pAb diluted 1/500 in 3% skim milk in TBS for 18 h at 4°C. Following 3 times washes with TBS, anti-mouse IgG alkaline phosphatase (KPL, USA) diluted 1:500 with 3% skim milk in TBS was added and incubated for 1 h at 37°C. The reactive proteins on nitrocellulose membrane were visualized by adding 5-bromo-4-chloro-3-indolyl phosphate/nitroblue tetrazolium (KPL, USA) substrate.

### Measurement of total IgG, IgG1, and IgG2a levels and study design

Total IgG, IgG1, and IgG2a responses of mice against crude unpurified and purified proteins of cystic fluid were measured by indirect biotin-streptavidin ELISA. 100 µl of crude cystic fluid (5 μg/ml) and purified proteins (1 μg/ml) were coated into wells of ELISA microtiter plates as above. Wells were then washed with PBS-T and blocked with 5% skim milk in PBS as described above. Pooled sera diluted 1:200 in PBS-T containing 3% skim milk were then added into wells and incubated for 1 h at 37°C. Biotinylated anti-mouse IgG1, IgG2a (eBioscience, UK), and IgG (KPL, USA) diluted 1/500 in 1% skim milk in PBS-T were then added into each well followed by streptavidin-HRP (KPL, USA). Finally, TMB (KPL, USA) substrate was added to the well. A proper washing procedure was conducted between each step as described above. The color development of TMB substrate was stopped by adding 50 μl of 1 N H_2_SO_4_ into each well. The OD of substrate was read by ELISA reader using 450 nm filter. The ELISA titers of IgG1, IgG2a, and total IgG were expressed as sample per positive ratio calculated according to the following formula [20].

ELISA ratio=(Sample OD-Negative OD)/(Positive OD-Negative OD)

In this study design, pre-sera collected before immunization was used as negative antibody control, total IgG levels were used as positive control of IgG response, and crude proteins were used as positive control of antigens.

## Results

### Immune response of mice against crude cystic fluid proteins of C. bovis

**Figure-1:**
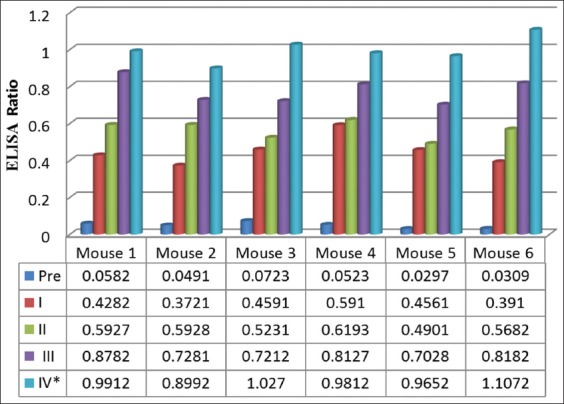
Immune responses of six mice against crude cystic fluid proteins of *Cysticercus bovis*. Antibody levels expressed as enzyme-linked immunosorbent assay ratios of six mice were shown. *Sera samples were collected 5 times, before the first immunization (Pre), and after the first (I), second (II), third (III), and fourth (IV) immunizations.

All six mice showed a similar immune response against crude cystic fluid proteins of *C. bovis*. Antibody against the parasite proteins was not detected in sera of mice collected before immunization. Low-to-moderate levels of antibody against cystic fluid proteins were detected after the first and the second immunizations. High levels of antibodies were detected after the third and the fourth immunization (Figure-1).

### Individual proteins isolated by SDS PAGE gel

**Figure-2:**
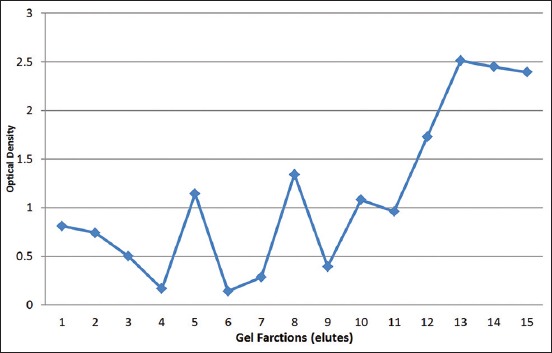
Optical density (OD) profiles of individual proteins eluted from gel cuts detected by enzyme-linked immunosorbent assay using polyclonal antibodies against crude cystic fluid of Cysticercus bovis. Note that the proteins with OD of higher than 0.5 were detected in elutes 1, 2, 5, 8, 10, 11, 12, 13, 14, and 15.

**Figure-3:**
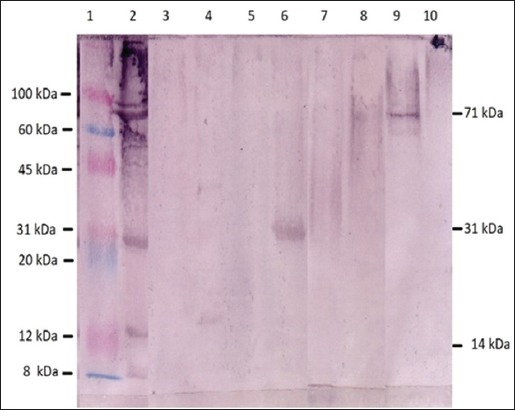
Reactive proteins from elutes of gel cuts detected by Western blotting assay using polyclonal antibodies against crude cystic fluid antigens of Cysticercus bovis. Lane 1: Molecular weight markers. Lane 2: Crude cystic fluid antigen. Lane 3-10: Elutes 3, 5, 7, 8, 12, 13, and 14, respectively.

Using large gel, 15 (1-15) horizontal gel cuts were made. Following elution and detection by ELISA using pAb, reactive proteins were detected in elutes of eight gel cuts (3, 5, 8, 11, 12, 13, 14, and 15) (Figure-2). The optical densities (ODs) of the eluted proteins varied from 0.93 to 2.53. By Western blotting using pAb, bands of single individual proteins were detected in elutes 5, 8, and 14 (Figure-3) with the molecular weights of 14, 31, and 71 KDa respectively. In crude cystic fluid, at least eight protein bands with molecular weights of 91, 71, 68, 51, 31, 18 14, and 8 KDa were observed (Figure-3).

### Profiles of IgG1 and IgG2a levels against p71, p31, and p14

**Figure-4:**
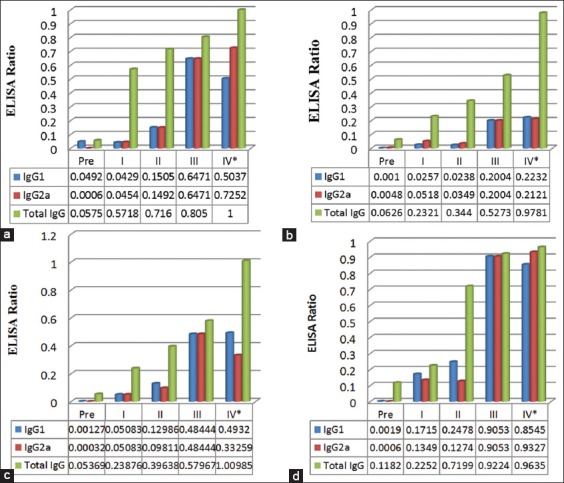
Profiles of immunoglobulin (Ig) G1, IgG2a, and total IgG levels induced by p71, p31, p14, and crude cystic fluid proteins of Cysticercus bovis in mice following 4 times immunizations with crude cystic fluid. Higher levels of IgG2a than IgG1 against p71 were detected following the fourth immunization (a). Balance levels of IgG1/IgG2a against p31 were detected following the third and the fourth immunizations (b). Higher levels of IgG1 than IgG2a against p14 were detected following the fourth immunization (c). Higher levels of IgG2a than IgG1 following the first and the second immunizations and a higher level of IgG1 than IgG2a following the fourth immunization were detected against crude cystic fluid proteins (d). *Sera samples were collected 5 times, before the first immunization (Pre), and after the first (I), second (II), third (III), and fourth (IV) immunizations.

In general, all three proteins induced both IgG1 and IgG2a responses in mice. No IgG1 and IgG2a antibodies against all three individual and crude cystic fluid proteins were observed in sera of mice before immunization (pre-sera). A balance of IgG1 and IgG2a immune response against the three proteins was observed following 3 times immunizations (first, second, and third) (Figure-4a-c). However, after the fourth immunization, different profiles of IgG1 and IgG2a responses were observed. IgG1 level induced by p71 declined after the fourth immunization, whereas IgG2a level increased (Figure-4a). In contrast, IgG1 level induced by p14 following the fourth immunization increased, whereas IgG2a level decreased (Figure-4c). The levels of IgG1 and IgG2a responses against p31 protein of *C. bovis* were generally low, and there was no difference between IgG1 and IgG2a levels following each of four immunizations (Figure-4b). Initially, higher IgG2a than IgG1 levels against crude cystic fluid proteins were observed following the first and the second immunizations, but later, higher IgG1 than IgG2a levels against crude cystic fluid proteins were observed following the fourth immunization (Figure-4d).

## Discussion

In this study, it was shown that crude cystic fluid proteins of *C. bovis* were immunogenic in mice as they induced antibody responses starting at the first immunization and increased until the fourth immunization. However, high levels antibody responses were detected only after the third and the fourth immunizations which indicate that high antibody responses against the parasite antigens only occur after prolonged exposure of mice with the parasite antigens. The result is in accord with the previous findings that the cystic fluid of *C. bovis* isolated from experimentally infected cattle contains immunogenic proteins [11]. In this study, at least eight protein bands were identified by mouse antisera in cystic fluid of *C. bovis* (Figure-3).

Although reactive proteins were detected in elutes of eight gel cuts by ELISA (Figure-2), only three showed single individual protein bands (elutes 5, 8, and 15) in Western blotting assay (Figure-3). Only those three individual proteins were, therefore, used for studying IgG1 and IgG2a responses of mice immunized with crude cystic proteins of *C. bovis*. The use of *C. bovis* proteins eluted from SDS-PAGE gel as antigens for ELISA and Western blotting assays has never been reported. However, a similar procedure has been used in the preparation antigen for ELISA and Western blotting assays of *Fasciola gigantica* excretory/secretory fluid [21]. The removal of SDS using acetone precipitation [18] appeared to be an important step in regaining the antigenicity of proteins eluted from SDS-PAGE gel.

In Western blotting assay using polyclonal antibodies, at least eight reactive protein bands were detected in crude cystic fluid of *C. bovis* which are similar to the previous findings by Dharmawan *et al*. [11] who detected seven immunogenic proteins in the cystic fluid of *C. bovis*. Meanwhile, using whole cystic proteins as antigens, Abuseir *et al*. [10] detected at least 10 proteins of *C. bovis* and most of the proteins cross-react with the proteins of another *Cysticercus* such as *Taenia granulosus* cyst and *Taenia hydatigena* cyst. Only two (p14 and 18) of the 10 proteins appear to be specific for *C. bovis* [10].

Following 4 times immunizations of mice, all three proteins, p71, p31, and p14, induced both IgG1 and IgG2a responses. However, following the fourth immunization, p71 tended to induce more IgG2a than IgG1 responses, whereas p14 appeared to induce more IgG1 than IgG2a responses. Meanwhile, p31 induced balance but low IgG1 and IgG2a responses following all four immunizations. As IgG2a level is the indicator of Th1 activation [22], p71 appears to be capable of inducing more cellular response than antibody response. By contrast, p14 appears to be capable of inducing more antibody response than cellular immune response as IgG1 level has been widely associated with the activation Th2 which plays important roles in the proliferation and differentiation of B cells into plasma cells [22]. In regard to p31, it appears that this protein induces low levels of both humoral and cellular immune responses. The profiles of IgG1/IgG2a levels against crude cystic fluid proteins of *C. bovis* are also interesting to note. Higher levels of IgG2a than IgG1 against crude cystic fluid proteins were observed following the first and the second immunizations, but then, higher levels of IgG1 than IgG2a against these proteins were observed following the fourth immunization (Figure-4d). The result may indicate that in the initial stage, crude cystic fluid proteins of *C. bovis* appear to induce more cellular than humoral immune responses, but in the later stage, the immune responses switch toward humoral immune response [5].

In mice, IL-4 secreted by Th2 promotes Ig class switching into IgG1 and Ig E and inhibits Ig class switching into IgG2a or IgG3. On the other hand, IFN-γ produced by Th1 promotes Ig class switching into IgG2a and IgG3 and inhibits Ig class switching into IgG1 [13]. It is, therefore, likely that p71 is a better inducer of both innate and adaptive cellular immune responses mediated by macrophages, NK cells, and cytotoxic T cells. Th1 produces IFN-γ which activates macrophages [23] and IL-2 which activates NK [24,25] and cytotoxic T cells [25]. Meanwhile, p14 is likely to play more roles in the proliferation of B cells into plasma cells to secrete antibody [26]. This finding was similar to the previous findings on *C. cellulosae* that different fractions of proteins were responsible for inducing cytokines for Th1 (IL-1β, tumor necrosis factor alpha, and IL-2 response) and Th2 (IL-4 and IL-10) activations [3].

The immunity produced against individual proteins/antigens of the *Cysticercu*s cystic fluid may kill the parasites or impairs their activities in the infected host. The immunity against p71 is likely to play important roles in cell-mediated killing and clearing parasites from the infected host by activating cellular immune responses such as those mediated by phagocytes [14,27], NK cells [28], and cytotoxic T lymphocytes [29,30]. Meanwhile, immunity against p14 appears to play roles in impairing the parasites activities in host through antibody-mediated response. Binding of antibody to parasite antigens can directly impair parasite activities [31] or facilitates phagocytosis by bringing the parasites in close contact with phagocytes [32,33].

## Conclusion

Of the three proteins studied, p71 is likely to be a good inducer of Th1 response, whereas p14 is the good inducer of Th2 response. The profiles of IgG1 and IgG2a levels against total crude cystic fluid proteins of *C. bovis* indicate that cellular immune responses against this parasite occur earlier than humoral immune response. Immunity against parasitic infections is a complex mechanism, and studies are still required to understand the roles individual proteins of *C. bovis* in modulating host immune responses.

## Authors’ Contributions

INMA conceived and designed the experiment, purified individual proteins, and examined immune response using ELISA and Western blotting assay. INMA also conducted result analysis and writing of the manuscript. IMD collected *C. bovis* from experimentally infected cattle and preparation of crude cystic fluid. IBMO took care of mice, carried out immunization, and collected sera from immunized mice. All authors read and approved the final manuscript.
